# A novel long non-coding RNA XLOC_004787, is associated with migration and promotes cancer cell proliferation by downregulating mir-203a-3p in gastric cancer

**DOI:** 10.1186/s12876-023-02912-2

**Published:** 2023-08-12

**Authors:** Renjie Miao, Zhendong Yao, Bingheng Hu, Tao Jin, Donglai Zhu, Yun Shi, Yuhua Gong, Shihe Shao, Chen Shao

**Affiliations:** 1 Department of Clinical Laboratory, Affiliated Third Hospital of Zhenjiang to Jiangsu University, Zhenjiang, 212001 Jiangsu China; 2 Department of Gastroenterology, The Affiliated Yixing Hospital of Jiangsu University, Yixing, 214200 Jiangsu China; 3 The Affiliated Hospital of Jiangsu University, Yizheng Road, Zhenjiang, 212013 Jiangsu China; 4 Department of Oncology, The Second Affiliated Hospital of Soochow University, Suzhou 215000, Jiangsu China

**Keywords:** Gastric cancer, Migration, Mir-203a-3p, Proliferation, XLOC_004787

## Abstract

**Background:**

Long noncoding RNAs (lncRNAs) have been identified as important regulatory factors implicated in a wide array of diseases, including various forms of cancer. However, the roles of most lncRNAs in the progression of gastric cancer (GC) remain largely unexplored. This study investigates the biological function and underlying mechanism of a novel lncRNA, XLOC_004787 in GC.

**Methods:**

The location of XLOC_004787 in GES-1 cells and HGC-27 cells were detected by fluorescence in situ hybridization (FISH) assay. The expression levels of XLOC_004787 were assessed using quantitative real-time fluorescence PCR (qRT-PCR) in various cell lines, including GES-1, MGC-803, MKN-45, BGC-823, SGC-7901, and HGC-27 cells. Functional assays such as Transwell migration, cell counting kit-8 (CCK-8), and colony formation experiments were employed to analyze the effects of XLOC_004787 and miR-203a-3p on cell migration and proliferation. Protein levels associated with GC in these cell lines were examined by Western blotting. The intracellular localization of β-catenin and P-Smad2/3 was assessed using immunofluorescence (IF) assay. Additionally, the interaction between XLOC_004787 and miR-203a-3p was investigated using a dual luciferase assay.

**Results:**

XLOC_004787 was localized at both the cytoplasm and nucleus of GES-1 cells and HGC-27 cells. Compared to normal tissues and GES-1 cells, XLOC_004787 expression was significantly upregulated in GC tissues and cells, with the highest and lowest expression observed in SGC-7901 and HGC-27 cells, respectively. Furthermore, a reduced expression of XLOC_004787 was seen to inhibit migration and proliferation in SGC-7901 cells. Western blotting analysis revealed that a decrease in XLOC_004787 expression correspondingly decreased the expression of N-cadherin, mmp2, mmp9, Snail, Vimentin, β-catenin, C-myc, Cyclin D1, and TGF-β, while concurrently increasing E-cadherin expression. This was also associated with diminished expression of P-Smad2/3 in relation to Smad2/3, and reduced P-Gsk3β expression in comparison to Gsk3β. Additionally, the nuclear entry of P-Smad2/3 and β-catenin was reduced by lower XLOC_004787 expression. Amplifying XLOC_004787 expression via pcDNA_XLOC_004787 suggested a potential for cancer promotion. Notably, XLOC_004787 was found to negatively regulate mir-203a-3p expression, with potential binding sites identified between the two. Higher mir-203a-3p expression was observed to decrease migration and proliferation, and enhance E-cadherin expression. Conversely, suppression of mir-203a-3p expression suggested a potential promotion of proliferation and migration in GC cells.

**Conclusions:**

These results suggest that XLOC_004787, found to be upregulated in GC tissues, potentially promotes proliferation and migration in GC cells. This occurs through the activation of TGF-β and Wnt/β-catenin signaling pathways and the expression of EMT-related proteins. Additionally, XLOC_004787 may influence cell migration and proliferation by modulating the signaling pathway via the adsorption and inhibition of mir-203a-3p.

## Introduction

Gastric cancer (GC), a malignancy primarily originating from the epithelium of the gastric mucosa, presents a significant global health challenge due to its intricate etiology and high mortality rate [1–4]. This complex disease is characterized by its close association with genetic mutations and irregular gene expression. Moreover, the pathogenesis and progression of GC have been directly linked to infections from certain viruses and microorganisms, most notably the Epstein-Barr virus (EBV) and *Helicobacter pylori* (*H. pylori*) [5–7]. On a global scale, GC ranks fifth in incidence and fourth in mortality rates [2], underlining the critical importance of investigating the molecular mechanisms underpinning its development. Understanding these mechanisms is pivotal for improving early diagnosis and therapeutic strategies for GC [8]. The disease is disproportionately prevalent in developing nations [9]. Notably, approximately half of these cases are found in Eastern Asia, with China representing a significant proportion. In fact, the incidence of GC in China constitutes 42.6% of the global total, while the mortality rate is at 45.0%, making China fifth in incidence and sixth in mortality among 183 nations [4, 10–13].

Long noncoding RNAs (lncRNAs), a category of noncoding RNAs exceeding 200 nucleotides in length that lack protein-coding capability, have emerged as critical players in a plethora of biological processes [14]. LncRNAs are involved in the regulation of gene expression, species evolution, embryonic development, metabolic processes, and even tumorigenesis. Certain lncRNAs are identified as potential tumor suppressors or promoters in various cancers, with their dysregulated expression often tied to the biological features of tumor cell proliferation, invasion, and metastasis [14, 15]. Moreover, lncRNAs have been implicated in a variety of other diseases, such as cardiovascular conditions, neurological disorders, and metabolic diseases, often as a result of abnormal expression patterns [16]. Recent studies have also demonstrated the involvement of specific lncRNAs in the immune system's biological processes, such as immune cell differentiation, cell cycle regulation, and apoptosis [17, 18]. Therefore, the potential to harness the regulatory capabilities of lncRNAs in treating various diseases represents a burgeoning area of research.

The intricate interplay between GC and lncRNAs pivots around the complex regulation of gene expression and diverse cellular processes [19]. Emerging evidence has implicated dysregulation of certain lncRNAs in the pathogenesis and progression of GC [20, 21]. This dysregulation appears to interfere with critical signaling pathways implicated in cellular differentiation and apoptosis. Moreover, a subset of these lncRNAs has shown potential as diagnostic and prognostic indicators for GC [22, 23]. However, a comprehensive understanding of the roles and therapeutic potential of lncRNAs in GC is yet to be fully elucidated. Our current study contributes to this knowledge base by identifying a novel lncRNA, XLOC_004787, demonstrating its significant upregulation in GC tissues and cell lines. Zhu H had reported that XLOC_004787 was aberrantly expressed in human gastric cells and tissues infected with *H. pylori* when compared to the control group, and the aberrant expression of XLOC_004787 may contribute to the pathological response and development of *H. pylori*-related diseases [24]. Yao M had also reported that XLOC_004787 was a upstream regulatory factor of miR-107 and was logically involved in inhibiting CVB3 replication and release, as well as the resulting inflammatory responses [25]. The development of gastric cancer is closely related to *H. pylori* infection. Therefore, we explored its functional role through both silencing and overexpression in GC cells. Our results indicate that XLOC_004787 plays a key role in cell migration and proliferation and impacts EMT, metastasis, proliferation, and the expression of various signaling pathway proteins. Furthermore, we provide evidence that XLOC_004787 may regulate the progression of GC by modulating the nuclear entry of P-Smad2/3 and β-catenin. An additional observation was a decrease in the expression of mir-203a-3p, suggesting XLOC_004787's role in mediating the migration and proliferation of GC cells. This is particularly significant as previous studies, such as those by Wang Z, et al., have reported that mir-203a-3p inhibits GC cell proliferation by targeting IGF-1R [26]. Therefore, our findings hint at the potential of XLOC_004787 as a novel therapeutic target for GC, necessitating further investigation in this promising area.

## Materials and methods

### Tumor tissue collection

In this study, both GC tissues and their corresponding non-tumor tissues were procured from the Affiliated People's Hospital of Jiangsu University during the period from 2017 to 2020. This study was conducted in strict accordance with the principles outlined in the Helsinki Declaration. All procedures pertaining to tissue sample collection and subsequent experimental protocols were duly approved by the Ethics Committee of Jiangsu University (Zhenjiang, China) and the Ethics Committee of the Affiliated People's Hospital, Jiangsu University. Post-collection, the GC samples were promptly immersed in TRIzol reagent and subsequently stored at -80 °C until further experimental use.

### Cell culture

This study utilized normal gastric mucosal epithelial cells (GES-1) in combination with five GC cell lines (BGC-823, HGC-27, MKN-45, SGC-7901, MGC-803). These cell lines were sourced from the Cell Bank of the Chinese Academy of Sciences (Shanghai, China) and the American Type Culture Collection (ATCC, Manassas, VA, USA), and were preserved in liquid nitrogen at the School of Medicine, Jiangsu University. The cells were cultured in Dulbecco's Modified Eagle Medium (DMEM; Gibco, Grand Island, NY, USA) enriched with 10% fetal bovine serum (FBS; Gibco). The cell culture process was carried out in a humidified incubator set at 37 °C and supplied with 5% CO_2_.

### Fluorescence in situ hybridization analysis (FISH) assay

Gene Pharma (Suzhou, China) was responsible for designing and synthesizing the XLOC_004787 probe conjugated with Cy3. The RNA-FISH kit was purchased from Gene Pharma. Cells were seeded onto glass coverslips, cultured overnight, and then fixed with 4% paraformaldehyde at room temperature for 30 min. After a 15-min treatment with 0.1% Triton X-100, cells were washed twice with PBS, and 200μL of 1 × hybridization buffer was added to each well, followed by incubation at 37 °C for 30 min. The liquid was discarded, and 200μL of 2 × buffer C was added and incubated at 37 °C for 30 min. The probe was diluted to 1 μM, denatured for 10 min in a water bath at 75 °C, and then 2μL of 1 μM biotin-labeled probe, 4μL of 1 μM SA-Cy3/FAM, and 14μL of PBS were added and incubated at 37 °C for 30 min. 180μL of buffer E was added afterwards. The cells were mixed with the probe mixture and incubated at 37 °C for 12–16 h for hybridization. The next day, cells were washed with 0.1% buffer F at 37 °C for 10 min, washed three times with 2 × buffer C, washed at 60 °C for 10 min each, and washed at 42 °C for 10 min each (three times in total). Finally, diluted DAPI-stained cells were added in the dark for 15 min, washed, sealed, and then observed with a fluorescence microscope (Leica, Mannheim, Germany).

### RNA Isolation and qRT-PCR Assays

The extraction of total RNA from GC cells and tissues was accomplished using the TRIzol reagent (Invitrogen, ThermoFisher Scientific), with the procedure executed in accordance with the provided guidelines.The isolated total RNA was then reverse transcribed into cDNA via the Superscript Reverse Transcriptase Kit (Vazyme, Nanjing, China). The real-time PCR amplification was performed using the ABI Step One Plus Real-Time PCR System (Applied Biosystems, Foster City, CA, USA), in conjunction with the TransStart Top Green qPCR Super Mix (TRAN, China). The primers for qRT-PCR were as follows:

- (1) GAPDH-Forward, 5′-AGGTGAAGGTCGGAGTCAAC-3′;
- GAPDH-Reverse, 5′-GGGTGGAATCATATTGGAACA-3′;
- (2) XLOC_004787-Forward, 5’-CTATTACTTTTCCTTTCAAGCACAC-3’;
- XLOC_004787-Reverse, 5′-TGACAAGTATTTGCTGAGTGCCTA-3’.

The qRT-PCR protocol included an initial step at 95 °C for 5 min, followed by 40 cycles of: 95 °C for 5 s; 60 °C for 20 s, 65 °C for 1 min, and 95 °C for 15 s. The 2^−ΔΔt^ method was subsequently employed for data quantification, designating GAPDH as the internal control.

### Cell transfection

Cell transfections were carried out using siRNA-XLOC_004787 (GenePharma, Shanghai, China) and pcDNA- XLOC_004787 (Sangon biotech, Shanghai, China), implemented with Lipofectamine 3000 (Invitrogen, ThermoFisher Scientific) following the manufacturer's recommended procedures. The efficiency of the cell transfections was subsequently evaluated using qRT-PCR. The siRNA-XLOC_004787 sequences used were as follows:

- (1) 5’-GCACACAUCUCAGUUGUUATT-3’,
- 3’-UAACAACUGAGAUGUGUGCTT-5’;
- (2) 5’-GCAACUCUUCACUUUACUATT-3’,
- 3’-UAGUAAAGUGAAGAGUUGCTT-5’;
- (3) 5’-GCUGCUCACACUCAUACUUTT-3’,
- 3’-AAGUAUGAGUGUGAGCAGCTT-5’.

### Colony formation and cell proliferation assay

The human gastric carcinoma cell line SGC-7901 was subjected to knockdown of XLOC_004787 via transfection with siRNA-XLOC_004787, while HGC-27 cells were transfected with pcDNA-XLOC_004787 plasmids and control plasmids. Post-transfection, the cells were detached using 0.25% trypsin, resuspended, and then enumerated. Subsequently, a population of 1 × 10^3^ cells was seeded into six-well plates and cultured under standard conditions, with the medium being refreshed every three days over a period of 10 to 14 days. After incubation, cells were fixed with 4% paraformaldehyde and stained with crystal violet. Additionally, 1 × 10^3^ cells were seeded into 96-well plates and maintained at 37 °C in a 5% CO_2_ atmosphere. The subsequent day, 10 μl CCK-8 reagent (Tongren, Shanghai, China) was added to each well at 24, 48, 72, and 96-h intervals and incubated for one hour. The absorbance of each well was recorded via enzyme-linked immunosorbent assay at a wavelength of 450 nm, with the resultant data averaged to plot a growth curve. This entire experimental procedure was performed in triplicate.

### Cell migration assay

The cell migration assays were performed using Transwell chambers (pore size, 8 µm; Corning, Costar, NY, USA). Both SGC-7901 and HGC-27 cells were transfected with siRNA-XLOC_004787, pcDNA-XLOC_004787, and corresponding controls, respectively. After 48 h, the cells were resuspended in serum-free DMEM medium and counted. A volume of 600 µL of medium containing 10% FBS was added into the lower chamber as a chemoattractant. Meanwhile, a total of 1 × 10^5^ cells in 200 µL of medium were uniformly seeded into the upper chamber. After incubation for 12 to 24 h, the chambers were immersed in 4% paraformaldehyde for 30 min at room temperature, rinsed with PBS, and the cells stained with crystal violet. The chamber was then washed again with PBS. Under a microscope, five random fields were chosen to image and count cells that migrated to the lower surface of the Transwell chamber membrane, for subsequent statistical analysis.

### Western blotting analysis and antibody utilization

SGC-7901 and HGC-27 cells were lysed utilizing RIPA lysis buffer (Beyotime Biotechnology, Shanghai, China) enriched with phenylmethanesulfonyl fluoride (PMSF) and phosphatase inhibitors. Uniform protein quantities (100 µg) were isolated using 10% SDS-PAGE gels, after which they were transferred onto PVDF membranes. Membranes were then blocked using 5% non-fat milk, and subsequently incubated with primary antibodies (as detailed in Table 1). Following a period of incubation, the membranes were washed and further incubated with HRP-conjugated anti-rabbit IgG (H + L) goat secondary antibodies (Fcmcs, Nanjing, China) for an hour under ambient conditions. Upon another washing cycle, protein detection was facilitated using the ECL system (Image Quant LAS 4000 mini, Pittsburgh, PA, USA) in accordance with the manufacturer's instructions.

**Table 1:** The antibodies used in this study

Names of antibodies	Dilution rate	Source of antibodies
E-cadherin	1:500	Wanleibio
N-cadherin	1:500	Wanleibio
mmp2	1:500	Wanleibio
mmp9	1:500	Wanleibio
Snail	1:500	Wanleibio
Vimentin	1:500	Wanleibio
β-catenin	1:500	Wanleibio
GSK3β	1:500	Wanleibio
P-GSK3β	1:400	Wanleibio
C-Myc	1:400	Wanleibio
Cyclin D1	1:500	Wanleibio
TGF-β	1:1000	Wanleibio
Smad2/3	1:500	Wanleibio
P-Smad2/3	1:400	Wanleibio
GAPDH	1:2000	Abcam

### Immunofluorescence analysis

Subsequent to transfection (48 h post-procedure), SGC-7901 and HGC-27 cells (both at a density of 2 × 10^4^) were dispersed into 24-well plates that contained cell slides. Following a period of 24 h post-inoculation, the cells were fixed using a 4% poly-methyl fermentation solution for 30 min and rinsed with PBS. Permeabilization of cells was achieved with 0.5% TritonX-100 (Sigma–Aldrich, Hong Kong, China) for a period of 10 min, after which the cells were rinsed and blocked using 5% BSA for 30 min. Primary antibodies for P-Smad2/3 and β-catenin were added, followed by overnight incubation at 4 °C. The following day, cells were rinsed with PBS, then treated with Cy3 labeled goat anti-rabbit IgG antibody (Huabio, Hangzhou, China) and incubated for 45 min at 37 °C in dark conditions. A final rinse with PBS was done before adding 0.5 ng/ml DAPI for 10 min. Upon washing with PBS, cells were sealed with an anti-fluorescence quencher, followed by observation under a confocal laser scanning microscope.

### Dual luciferase reporter assay

GenePharma Co. (Suzhou, China) constructed both the wild type (WT) and mutant (MUT) XLOC_004787 plasmids. Human embryonic kidney (HEK 293 T) cells, at a density of 1.0 × 10^5^ cells/well, were cultured in a 24-well plate. Subsequently, the cells were co-transfected with either the WT or MUT plasmids and mir-203a-3p mimics (GenePharma, Shanghai, China) using Lipofectamine 3000 as a transfection reagent. After a co-transfection period of 36 h, both renilla and firefly luciferase activities were quantified. The ratio of these activities was then used to evaluate the interaction between XLOC_004787 and mir-203a-3p.

### Statistical analysis

Statistical analyses for this research were conducted using GraphPad Prism 8.2 software (La Jolla, USA). Image Pro Plus software (Media Cybernetics, USA) was used to obtain the relative gray-scale value of the bands and to perform cell counting. Independent *t*-tests were utilized for comparisons among different groups. Each experiment was independently conducted three times. All statistical tests were two-tailed, with a threshold of *p* < 0.05 set as the criteria for statistical significance.

## Results

### XLOC_004787 is highly expressed both in GC tissues and cells

**Fig. 1:**
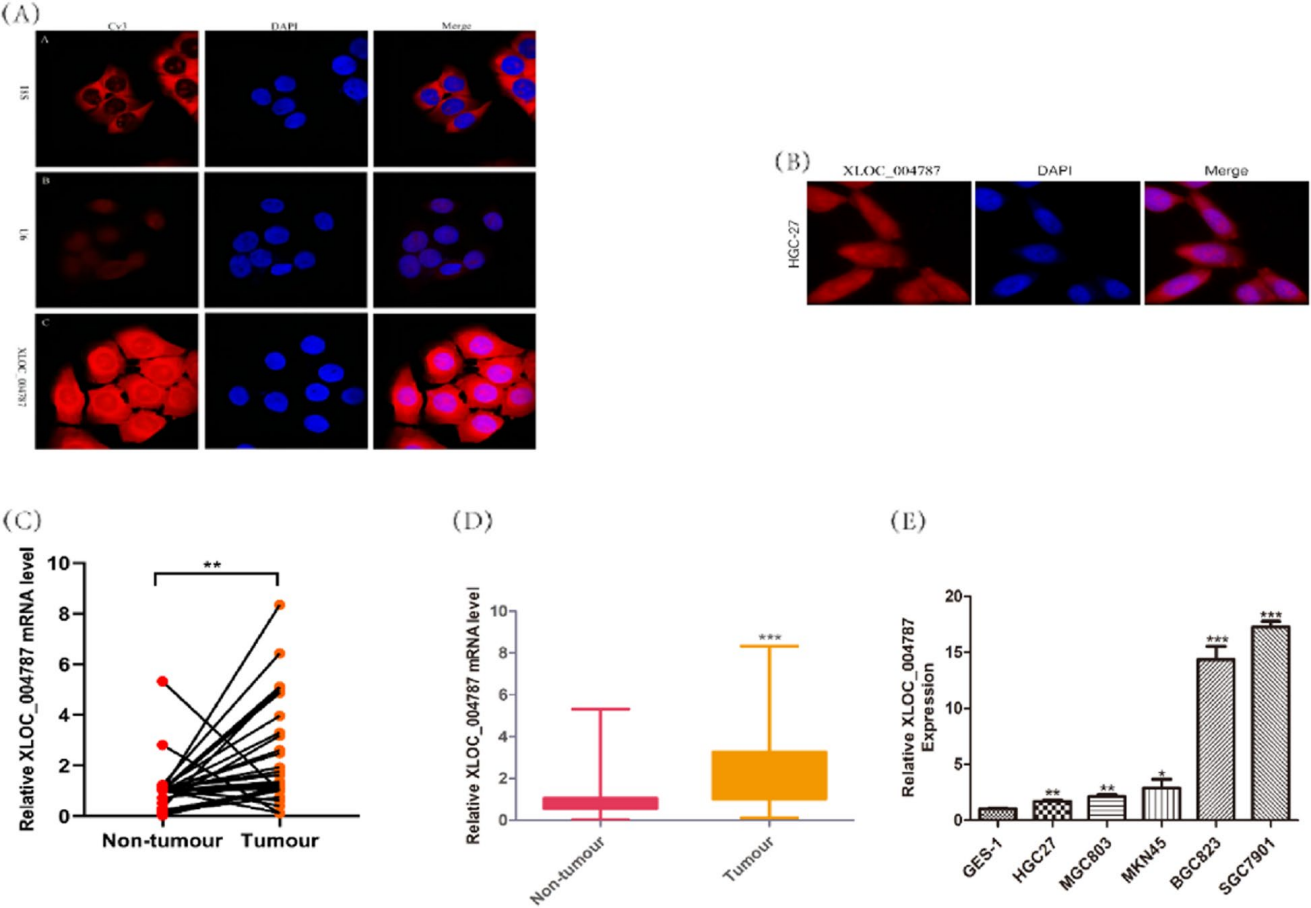
XLOC_004787 is upregulated both in GC tissues and cells. **A** and **B** RNA-FISH assay revealed the cytoplasmic and nuclear location of XLOC_004787 in GES-1 cells and HGC-27 cells (original magnification × 600). **C** and **D** XLOC_004787 mRNA expression in 32 paired GC tissues and the adjacent normal tissues by qRT-PCR.** E** qRT-PCR detected XLOC_004787 levels within GES-1 cells as well as five GC cell lines. *P < 0.05, **P < 0.01, ***P < 0.001

To determine the location of XLOC_004787, according to RNA-FISH assay, XLOC_004787 was localized at both the cytoplasm and nucleus of GES-1 cells and HGC-27 cells (Fig. 1A, B). The expression level of XLOC_004787 mRNA was measured in 32 pairs of fresh frozen GC tissues and matched normal gastric tissues. The results showed that XLOC_004787 was upregulated in GC samples (Fig. 1C, D). In addition, XLOC_004787 exhibited high expression levels in vitro gastric cancer cell lines, with SGC-7901 cells showing the highest expression and HGC-27 cells showing the lowest expression compared to GES-1 cells (Fig. 1E). Based on these findings, XLOC_004787 is expressed at high levels in both GC tissues and cells.

### XLOC_004787 overexpression promotes GC cells proliferation and migration

**Fig. 2:**
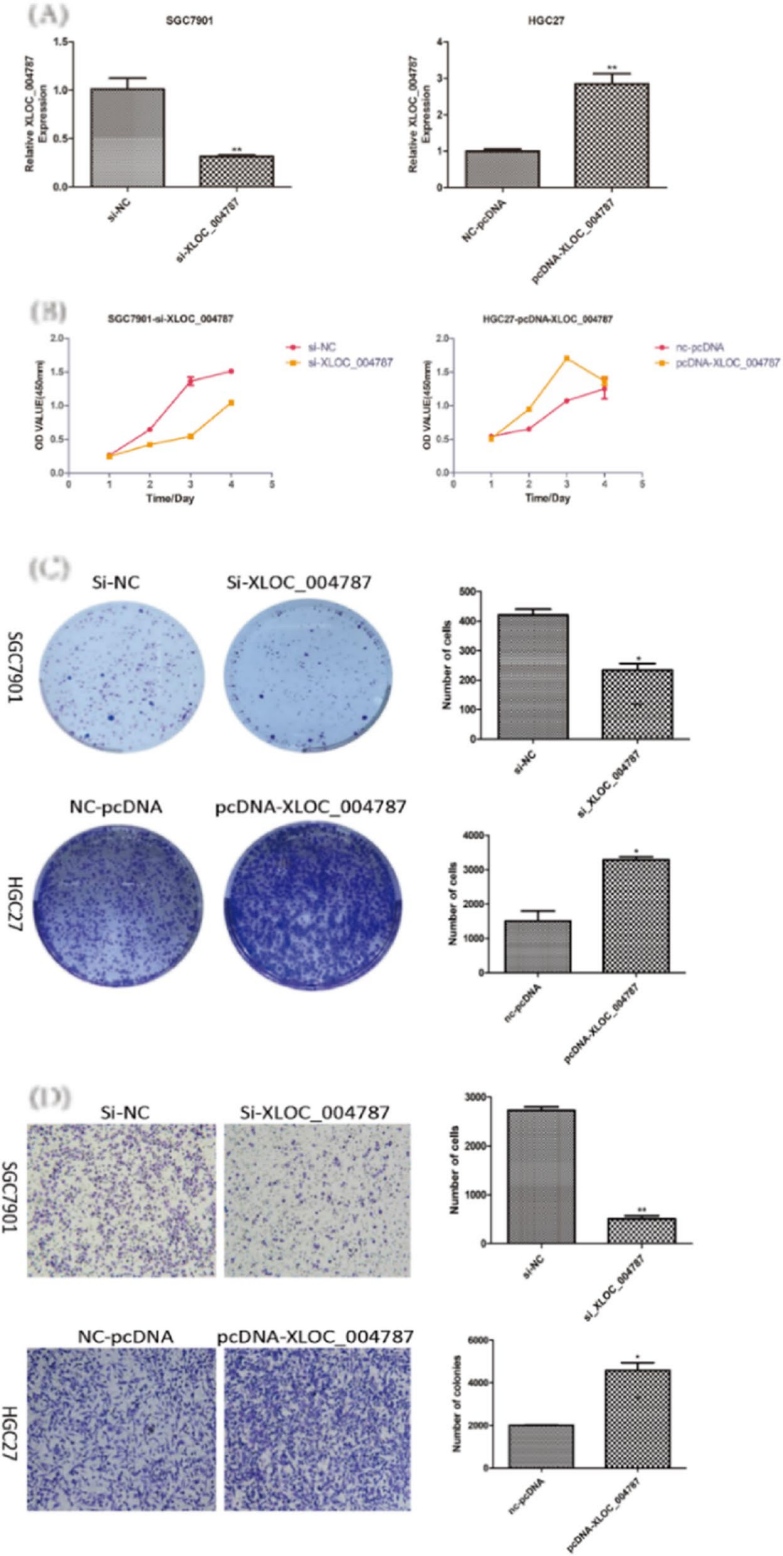
High expression of XLOC_004787 promoted cell migration and proliferation in GC cells. **A** qRT-PCR was used to detect the efficiency of knockdown and overexpression of XLOC_004787. **B** CCK-8 assay was conducted to observe the ability of proliferation in GC cells. **C** Colony-formation assay was carried out to observe the ability of proliferation in GC cells. **D** Transwell migration assay was used to detect the efficiency of cell migration (original magnification × 40). *P < 0.05, **P < 0.01, ***P < 0.001

The expression of XLOC_004787 was knocked down by siRNA in SGC-7901 cells (Fig. 2A). The expression of XLOC_004787 was overexpressed by pcDNA-XLOC_004787 in HGC-27 cells (Fig. 2A). To explore the function of XLOC_004787 in GC cells, CCK-8 proliferation experiments, plate clone formation experiments and transwell chamber migration experiments found that over-expressed XLOC_004787 in HGC-27 cells promoted cell growth ability and cell migration ability, the HGC-27 cell clones became larger and the number increased compared to control group. (Fig. 2B-D). Siliencing XLOC_004787 markedly weakened the proliferation and migration in SGC-7901 cells, the SGC-7901 cell clones became smaller than the control cells, the clones number decreased. (Fig. 2B-D).

### XLOC_004787 induces proliferation and migration in GC cells by regulating EMT-related proteins, Wnt/β-Catenin signaling pathway and TGF-β signaling pathway

**Fig. 3:**
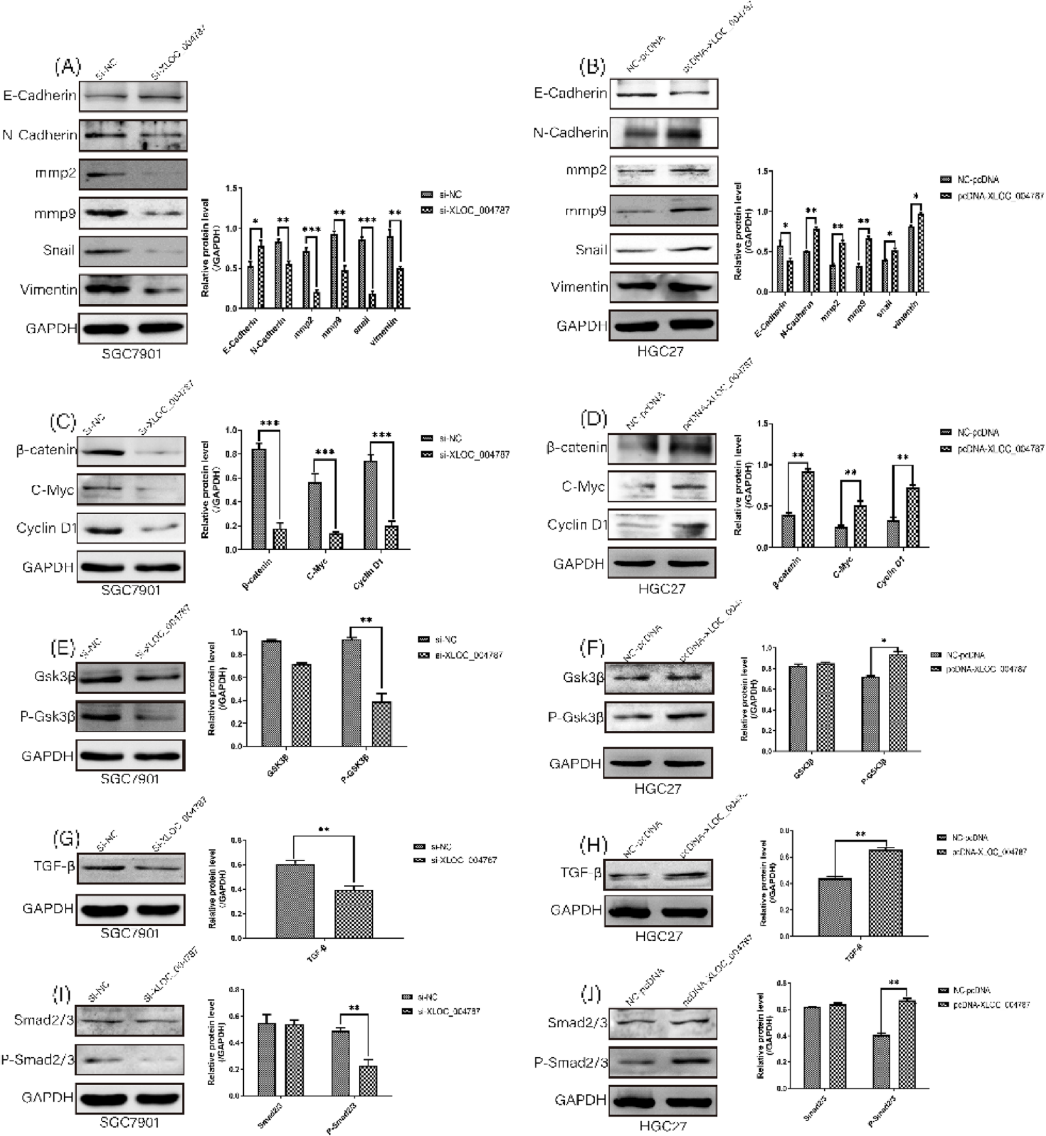
High expression of XLOC_004787 upregulated the proteins levels related with migration, proliferation and EMT in GC cells. **A, C** and **G** By western blotting analysis, the level of E-cadherin increased and the standard of mmp9, Snail, Vimentin, β-catenin, C-myc, Cyclin D1 and TGF-β decreased after XLOC_004787 knockdown in SGC-7901 cells. **B, D** and **H** By western blotting detection, the standard of E-cadherin declined and the level of mmp9, Snail, Vimentin, β-catenin, C-myc, Cyclin D1 and TGF-β raised after XLOC_004787 overexpression in HGC-27 cells. **E** and **I** Western blotting found that knock down XLOC_004787 in SGC-7901 cells reduced the expression of P-Gsk3β and P-Smad2/3 relative to Gsk3β and Smad2/3. **F** and** J** Western blotting showed that upregulated XLOC_004787 in HGC-27 cells enhanced the expression of P-Gsk3β and P-Smad2/3 relative to Gsk3β and Smad2/3. Data were shown as mean ± SEM. The experiments were repeated at least three times. *P < 0.05, **P < 0.01, ***P < 0.001

In the following step, EMT-related proteins, Wnt/β-Catenin signaling pathway and TGF-β signaling pathway which related to proliferation and migration were detected by Western blotting (Fig. 3A-J). On the one hand, the level of N-Cadherin, mmp2, mmp9, Snail, Vimentin, β-catenin, C-myc, Cyclin D1 and TGF-β obviously declined while the expression of E-Cadherin increased in the XLOC_004787 knockdown group. On the other hand, it showed the the opposite result after the XLOC_004787 overexpression (Fig. 3A-D, G and H). Furthermore, after XLOC_004787 knocked down, compared to control group the expression of P-Gsk3β and P-Smad2/3 significantly decreased relative to Gsk3β and Smad2/3. (Fig. 3E, I). Then the expression of P-Gsk3β and P-Smad2/3 markedly increased relative to Gsk3β and Smad2/3 after the XLOC_004787 overexpression (Fig. 3F, J).

### XLOC_004787 induces proliferation and migration by regulating the efficiency of entering the nucleus of P- Smad2/3 and β-catenin

**Fig. 4:**
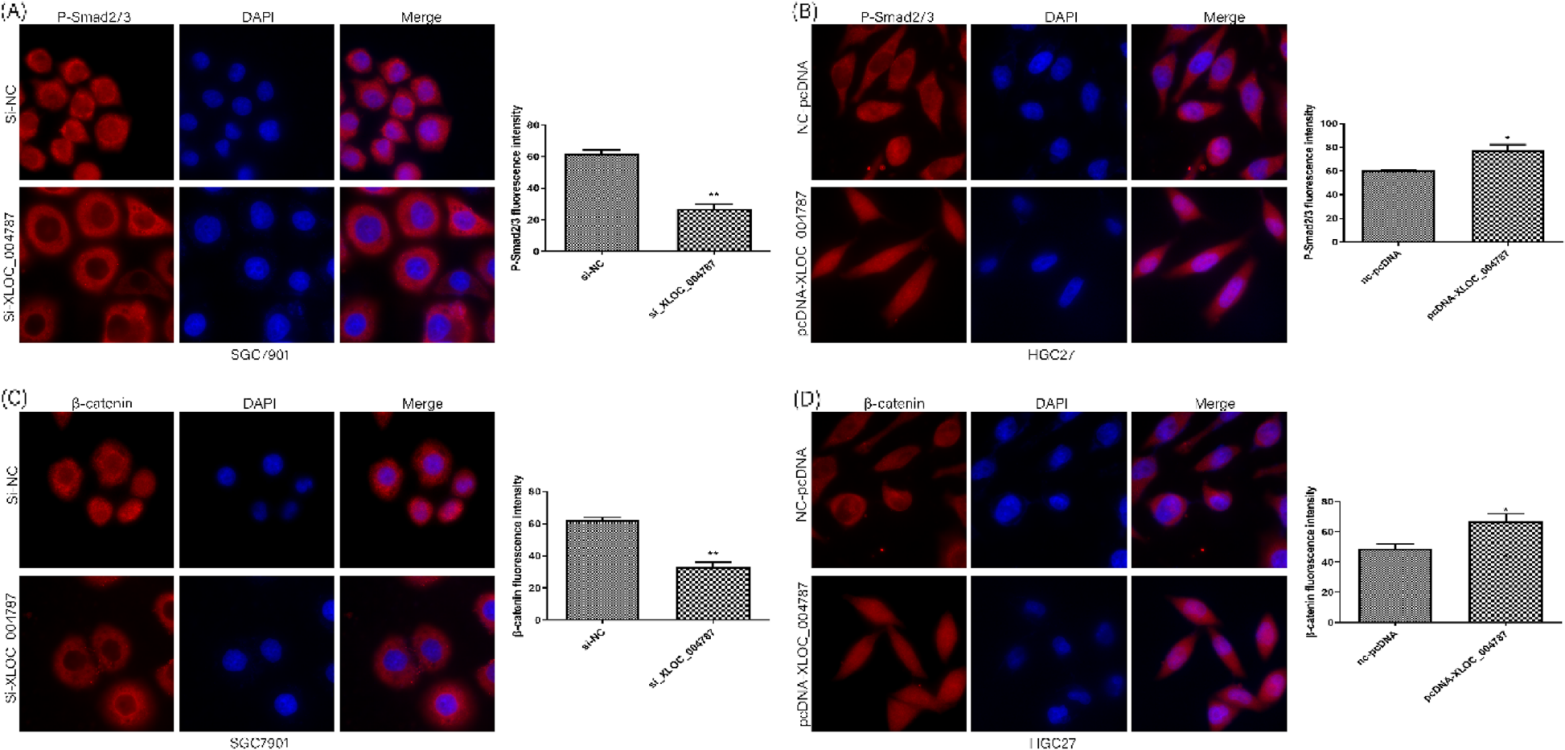
High expression of XLOC_004787 promoted the efficiency of entering the nucleus of P- Smad2/3 and β-catenin. **A** and **C,** The amount of P-Smad2/3 and β-catenin entering the nucleus is reduced after XLOC_004787 silencing by IF, scale bar = 25 µm. **B** and **D,** The quantity of P-Smad2/3 and β-catenin entering the nucleus is enhanced after XLOC_004787 overexpression by IF (original magnification × 600)

On the one hand, IF suggested that that the amount of P-Smad2/3 and β-catenin entering the nucleus is reduced when we knocked down the expression of XLOC_004787 in SGC-7901 cells (Fig. 4A, C). On the other hand, IF found that the efficiencey of P-Smad2/3 and β-catenin entering the nucleus is enhanced after XLOC_004787 overexpression in HGC-27 cells (Fig. 4B, D).

### XLOC_004787 decreases mir-203a-3p mRNA expression

**Fig. 5:**
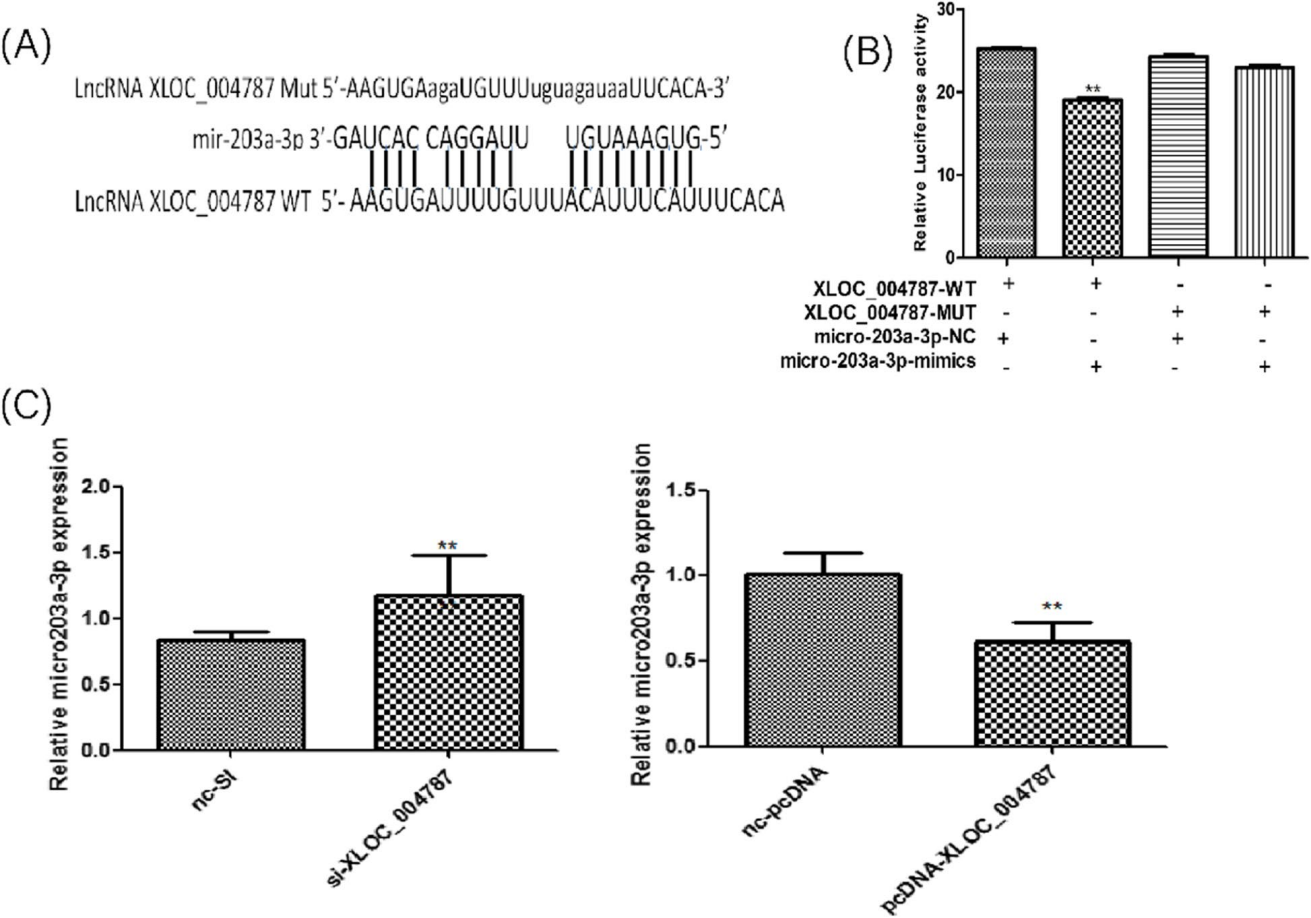
XLOC_004787 has an inhibitory effect on the expression of mir-203a-3p. **A** Through the prediction of AnnoLnc, mir-203a-3p had binding site with XLOC_004787. **B** Fluorescence had been inhibited after transfection of the wild type plasmids, fluorescence inhibition is released after transfection of the mutant plasmid. **C** mir-203a-3p expression increased after XLOC_004787 knocked down, mir-203a-3p expression decreased after XLOC_004787 overexpressed by qRT-PCR analysis. *P < 0.05, **P < 0.01, ***P < 0.001

AnnoLnc (http://annolnc.cbi.pku.edu.cn/) was used to predict the downstream miRNAs of XLOC_004787 and found the mir-203a-3p had binding sites with XLOC_004787 (Fig. 5A). Dual luciferase reporter assay demonstrated that wild type plasmids of XLOC_004787 co-transfected with mir-203a-3p mimics decreased the value of luciferase ratio while and mutant plasmids has no significant (Fig. 5B). In addition, mir-203a-3p expression increased after XLOC_004787 knockdown in HGC-27 cells, mir-203a-3p expression decreased after XLOC_004787 overexpression (Fig. 5C). Wang Z has reported that miR-203a-3p function as a tumor suppressor by targeting IGF-1R in GC [9]. This suggested that XLOC_004787 may mediated miR-203a-3p/IGF-1R influenced the progression of GC.

### mir-203a-3p overexpression inhibits GC cells proliferation and migration

**Fig. 6:**
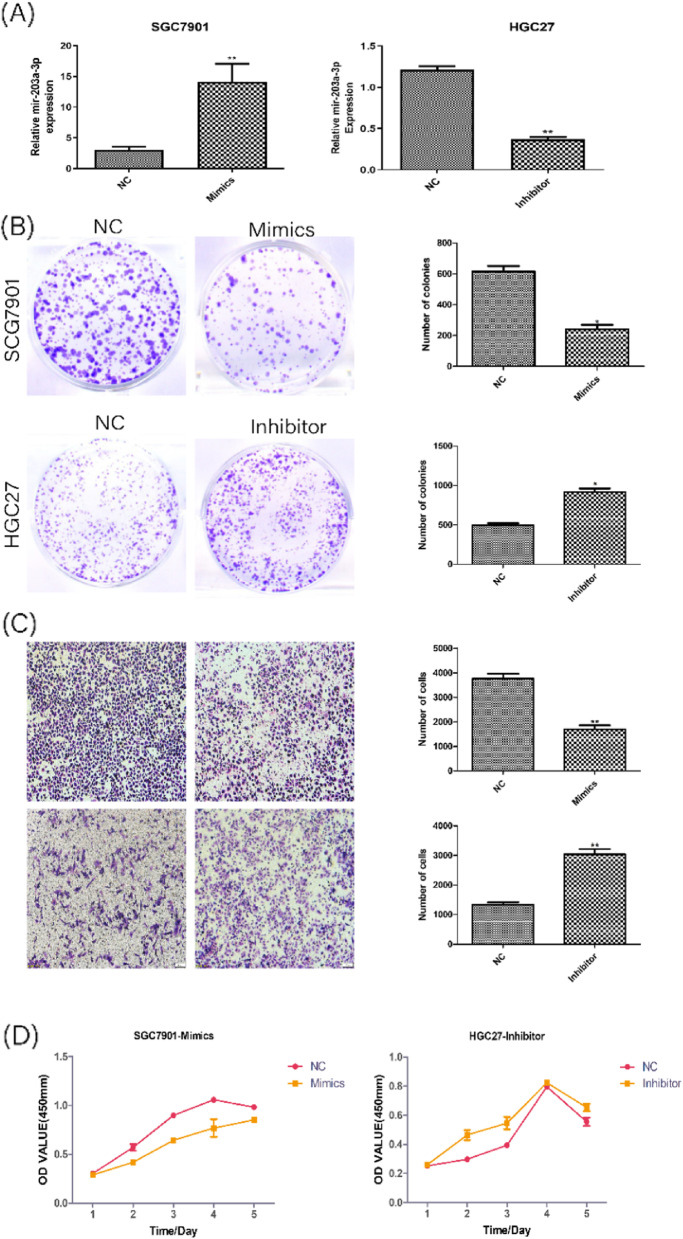
High expression of mir-203a-3p suppressed cell migration and proliferation in GC cells. **A** qRT-PCR was used to detect the efficiency of knockdown and overexpression of mir-203a-3p. **B** Colony-formation assay was carried out to observe the ability of proliferation in GC cells. **C** Transwell migration assay was used to detect the efficiency of cell migration (original magnification × 40). **D** CCK-8 assay was conducted to observe the ability of proliferation in GC cells. *P < 0.05, **P < 0.01, ***P < 0.001

The expression of mir-203a-3p was overexpressed by mimics in SGC-7901 cells (Fig. 6A). The expression of mir-203a-3p was knocked down by inhibitor in HGC-27 cells (Fig. 6A). To explore the function of mir-203a-3p in GC cells, CCK-8 proliferation experiments, plate clone formation experiments and transwell chamber migration experiments found that over-expressed mir-203a-3p in SGC-7901 cells suppressed cell growth ability and cell migration ability, the SGC-7901 cell clones became smaller and the number decreased compared to control group. (Fig. 6B-D). Inhibiting mir-203a-3p markedly enhanced the proliferation and migration in HGC-27 cells, the HGC-27 cell clones became larger than the control cells, the clones number increased (Fig. 6B-D).

### mir-203a-3p affects proliferation and migration in GC cells by regulating EMT-related proteins, Wnt/β-Catenin signaling pathway and TGF-β signaling pathway

**Fig. 7:**
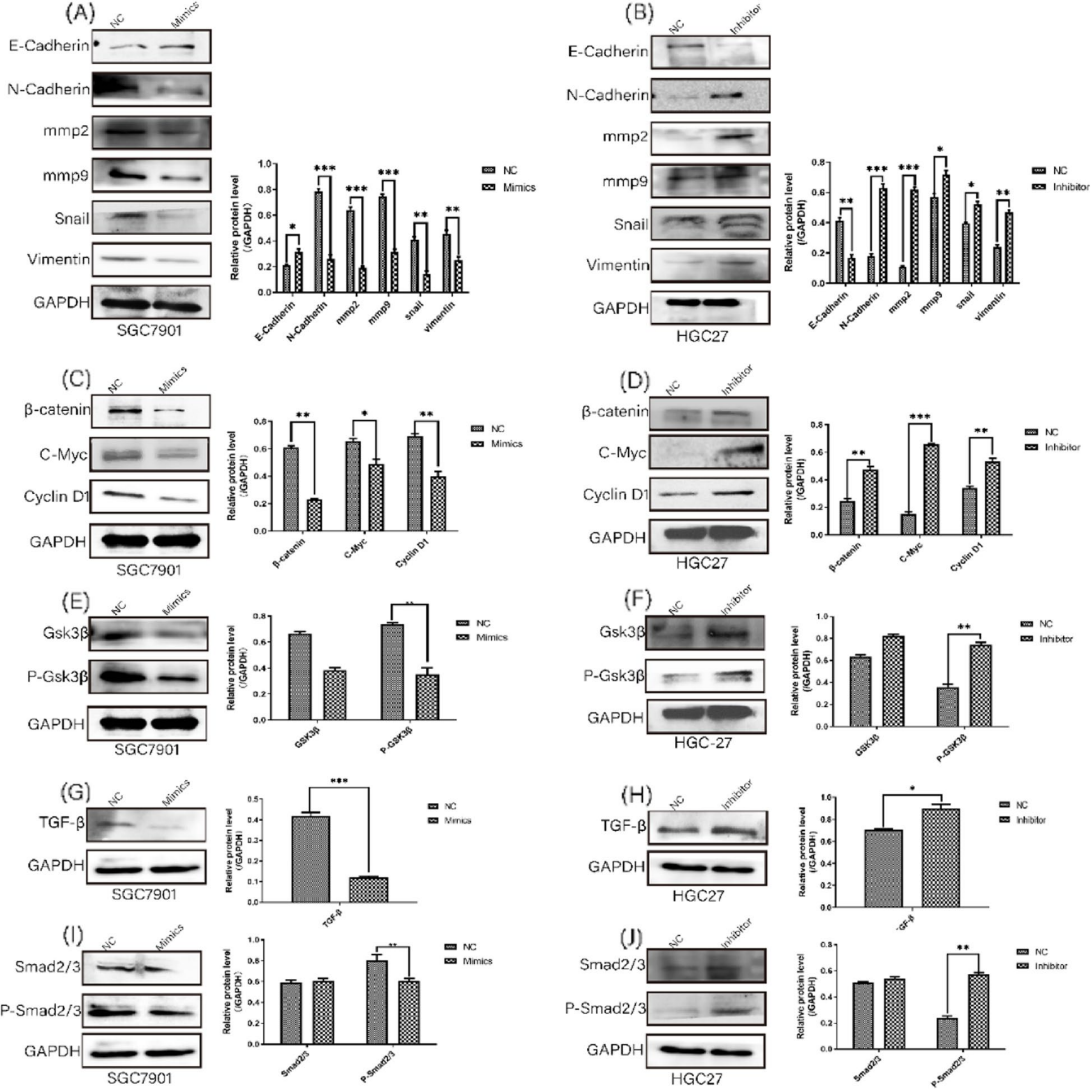
High expression of mir-203a-3p suppressed the proteins levels related with migration, proliferation and EMT in GC cells. **A, C** and **G** By western blotting analysis, the level of E-cadherin increased and the standard of mmp9, Snail, Vimentin, β-catenin, C-myc, Cyclin D1 and TGF-β decreased after mir-203a-3p overexpression in SGC-7901 cells. B, D and H By western blotting detection, the standard of E-cadherin declined and the level of mmp9, Snail, Vimentin, β-catenin, C-myc, Cyclin D1 and TGF-β raised after mir-203a-3p inhibition in HGC-27 cells. E and I Western blotting found that upregulated mir-203a-3p in SGC-7901 cells reduced the expression of P-Gsk3β and P-Smad2/3 relative to Gsk3β and Smad2/3. F and J Western blotting showed that inhibited XLOC_004787 in HGC-27 cells enhanced the expression of P-Gsk3β and P-Smad2/3 relative to Gsk3β and Smad2/3. Data were shown as mean ± SEM. The experiments were repeated at least three times. *P < 0.05, **P < 0.01, ***P < 0.001

In the following step, EMT-related proteins, Wnt/β-Catenin signaling pathway and TGF-β signaling pathway which related to proliferation and migration were detected by Western blotting (Fig. 7A-J). On the one hand, the level of N-Cadherin, mmp2, mmp9, Snail, Vimentin, β-catenin, C-myc, Cyclin D1 and TGF-β obviously declined while the expression of E-Cadherin increased in the mir-203a-3p mimics group. On the other hand, it showed the the opposite result after the mir-203a-3p inhibition (Fig. 7A-D, G and H). Furthermore, after mir-203a-3p knocked down, compared to control group the expression of P-Gsk3β and P-Smad2/3 significantly increased relative to Gsk3β and Smad2/3(Fig. 7F, J). Then the expression of P-Gsk3β and P-Smad2/3 markedly decreased relative to Gsk3β and Smad2/3 after the mir-203a-3p overexpression (Fig. 7E, I).

## Discussion

The interplay between lncRNAs and various diseases, particularly cancers, is intricate and multifaceted [16, 18]. A surge of research within the last decade has unveiled the dual role of lncRNAs, acting as potential oncogenes or tumor suppressors in the development of cancer [27]. Their dysregulation is often associated with pivotal biological characteristics including tumor cell proliferation, invasion, and metastasis [18]. Specific lncRNAs such as MALAT1, HOTAIR, and UCA1, are found to be highly expressed in GC and are implicated in lymph node metastasis, pathological grading, and recurrence rates [28–30]. Conversely, lncRNAs like SNHG5 and LINC00675 are known to behave as suppressors of GC, with their diminished expression linked to an increase in GC cell proliferation, invasion, and metastasis [31, 32]. While we have gained substantial insight into lncRNA regulatory mechanisms and their various biological roles in the human body, the discovery and characterization of novel functional lncRNAs remain a vibrant area of research. In this study, we identified a novel lncRNA, XLOC_004787, through chip-based screening and subsequently verified its full length using sequencing techniques. Previous studies on XLOC_004787 have focused on its role in certain viruses, such as the Coxsackie B3 virus (CVB3) [25], but its function and specific involvement in GC remain unclear. Our findings indicate elevated expression of XLOC_004787 in both GC tissues and cell lines. Moreover, we observed that an upregulated expression of XLOC_004787 in HGC-27 cells promoted cell migration and proliferation. Conversely, the suppression of XLOC_004787 in SGC-7901 cells led to the inhibition of these same processes.

The heightened expression of XLOC_004787 could potentially modulate mmp9 and mmp2, thereby influencing the migratory patterns and proliferation of GC cells. We observed the phenomenon of EMT in numerous malignant tumors, which gradually morphs tightly bound epithelial cells into more invasive mesenchymal cells. It should be noted that early onset of EMT in tumors has the potential to modify the tumor cell microenvironment, including factors like inflammation, fibrosis, and neovascularization [33]. In malignant tumors, alterations in epithelial cells are typically marked by the reduction of E-cadherin, an epithelial cell marker protein, coupled with an increase in the expression of N-cadherin, Snail, and Vimentin [33, 34]. Our findings indicate that overexpression of XLOC_004787 enhances the levels of N-cadherin, Snail, and Vimentin, while concurrently lowering the expression of E-cadherin in HGC-27 cells.

Additionally, the TGF-β signaling pathway, known to influence cell cycle, differentiation, extracellular matrix synthesis, and tumor immune response, plays a pivotal role [35]. The Wnt/β-catenin signaling pathway has been demonstrated to be integral in embryonic development, tissue regeneration, and particularly in cancer development. Aberrant expression of the Wnt/β-catenin signaling pathway has been associated with various cancers, including colon, breast, and liver cancers [36, 37]. This abnormal activation of the Wnt/β-catenin signaling pathway can foster proliferation, invasion, and metastasis of cancer cells during cancer progression [38]. Our results show that XLOC_004787 may mediate the proliferation of GC cells via the TGF-β and Wnt/β-catenin signaling pathways. The findings of our study revealed that knockdown of XLOC_004787 substantially attenuated the migration, proliferation, and EMT of GC cells. Conversely, overexpression of XLOC_004787 resulted in an inverse outcome. In HGC-27 cells overexpressing XLOC_004787, the concentration of TGF-β, P-Smad2/3, C-myc, CyclinD1, P-GSK3β, and β-catenin increased, with higher nuclear localization of P-Smad2/3 and β-catenin. In contrast, in SGC-7901 cells exhibiting downregulated XLOC_004787 expression, the levels of TGF-β, P-Smad2/3, C-myc, CyclinD1, P-GSK3β, and β-catenin diminished, and P-Smad2/3 and β-catenin were less abundant in the nucleus.

In the human body, the regulation of lncRNAs operates within an intricate network, and their interaction with microRNAs (miRNAs) is multifaceted. LncRNAs have been observed to function as “miRNA sponges [27],” serving to absorb and thereby modulate miRNA expression—an interaction denoted as the “miRNA sponge effect.” This interaction impedes miRNA binding to their intended target RNAs, thereby influencing their expression [39]. Conversely, miRNAs are capable of altering the expression and function of lncRNAs by influencing their transcription or splicing processes [40]. In the scope of this study, it was predicted and substantiated that the lncRNA XLOC_004787 targets the downstream miRNA, miR-203a-3p. The results showed that XLOC_004787 and miR-203a-3p share sequence complementarity and exhibit reciprocal regulation. Upon overexpressing miR-203a-3p, we noted a reduction in GC cell proliferation and migration, accompanied by the inhibited expression of EMT-related proteins, the TGF-β signaling pathway, and the Wnt/β-catenin signaling pathway. Simultaneously, there was an augmentation in E-cadherin expression. Conversely, the inhibition of miR-203a-3p produced the inverse results. Mir-203a-3p has been previously reported as a tumor suppressor gene targeting IGF-1R [26], thus this study further clarifies this relationship.

The main limitations of this study are its small inclusion of clinical subjects and the lack of in vivo experiments due to existing laboratory and equipment constraints. For a more comprehensive understanding of this lncRNA's applicability as a potential biomarker, it would be prudent to involve larger patient groups in subsequent clinical trials. Furthermore, the application of inhibitors or inducers related to the Wnt/β-catenin and TGF-β signaling pathways could further elucidate the role of XLOC_004787 in promoting GC cell proliferation and migration.

## Conclusion

In summary, our findings suggest that the overexpression of XLOC_004787 facilitates GC cell migration and proliferation via the mediation of EMT-related proteins, the TGF-β signaling pathway, and the Wnt/β-catenin signaling pathway. Hence, it holds potential as a novel diagnostic and therapeutic marker for GC patients.

## Data Availability

The datasets generated and/or analyzed during the current study are not publicly available, due to the privacy of the enrolled subjects, but may be provided by the corresponding author upon reasonable request.

## Abbreviations

GC: Gastric cancer
LncRNAs: Long noncoding RNAs
MicroRNA: miRNA
FISH: Fluorescence in situ hybridization analysis
qRT-PCR: Quantitative real-time fluorescence PCR
CCK-8: Cell counting kit-8
IF: Immunofluorescence
TGF-β: Transforming growth factor-β
EMT: Epithelial mesenchymal transformation
H.pylori: Helicobacter pylori
PMSF: Phenylmethanesulfonyl fluoride
RIPA: Radio immunoprecipitation
